# Effect of Quaternary Ammonium Salts with Fluorine Atoms on Selected Weed Species

**DOI:** 10.1007/s00128-017-2033-6

**Published:** 2017-02-14

**Authors:** Robert Biczak, Barbara Pawłowska, Maciej Płatkowski, Michał Stręk, Arkadiusz Telesiński

**Affiliations:** 1 Department of Biochemistry and Ecotoxicology, The Faculty of Mathematics and Natural Sciences, Jan Długosz University in Częstochowa, Armii Krajowej Av. 13/15, 42-200 Częstochowa, Poland; 2 Department of Plant Physiology and Biochemistry, Faculty of Environmental Management and Agriculture, West Pomeranian University of Technology in Szczecin, Juliusza Słowackiego st. 17, 71-434 Szczecin, Poland

**Keywords:** Phytotoxicity, Dry weight, Chlorophyll, Inhibition of plant growth and roots

## Abstract

This study investigated the effects of four structurally different quaternary ammonium salts (QASs), i.e., tetrabutylammonium tetrafluoroborate [TBA][BF_4_], tetrahexylammonium tetrafluoroborate [THA][BF_4_], tetrabutylammonium hexafluorophosphate [TBA][PF_6_], and tetrahexylammonium hexafluorophosphate [THA][PF_6_], on the growth and development of three weed species: gallant soldier (*Galinsoga parviflora* Cav.), white goosefoot (*Chenopodium album* L.) and common sorrel (*Rumex acetosa* L.). The examined compounds were applied in the form of foliar spraying and soil application. Strong herbicidal properties of the examined compounds were demonstrated in case of their soil application. Growth inhibition of plant shoots and roots was greater with soil application than with foliar treatment. The strongest herbicidal activity of compounds was demonstrated with [TBA][BF_4_] have demonstrated [TBA][BF_4_] and [TBA][PF_6_] applied to the soil, while [THA][BF_4_] demonstrated the weakest herbicidal action. The increased concentration of applied QASs caused a decrease in the assimilation pigments, change in dry weight content and inhibition of length of shoots and roots.

In recent years, much importance has been ascribed to the discovery of environmentally safe herbicides in crop protection (Praczyk and Skrzypczak 2004; Travlos et al. 2014). With this in mind, research teams from around the world have been looking for new compounds that exhibit desirable herbicidal properties and are concurrently harmless to other organisms. An example of a new and promising class of compounds with herbicidal properties is that of the herbicidal ionic liquids (HILs) (Pernak et al. 2016; Niemczak et al. 2015).

Quaternary ammonium salts (QASs) are a group of compounds within the class that have demonstrated numerous desirable properties, including wetting, emulsifying, dispersing, antistatic and preservative properties. Moreover, they have also demonstrated biological activity (e.g., algaecidal, fungicidal, and bactericidal), resulting in a wide range of practical applications (Grabińska-Sota 2004). Representatives of this group of compounds include tetrabutylammonium tetrafluoroborate [TBA][BF_4_], tetrahexylammonium tetrafluoroborate [THA][BF_4_], tetrabutylammonium hexafluorophosphate [TBA][PF_6_], and tetrahexylammonium hexafluorophosphate [THA][PF_6_]. These compounds are insoluble in water, but well soluble in organic solvents. They are relatively inexpensive compared to other QASs or ionic liquids (ILs). The use of compounds that are already commercially available for testing as potential herbicides eliminates the costs associated with the synthesis and purification of new compounds.

This study examined the effects of [TBA][BF_4_], [THA][BF_4_], [TBA][PF_6_] and [THA][PF_6_] on the growth and development of three commonly occurring weed species: *G. parviflora, C. album* and *R. acetosa*. The new groups of biologically active compounds which would exhibit selective or total herbicidal properties, concurrently being non-toxic for the environment, have been searched during this study. Such compounds could be used in the future as alternatives to currently used herbicides, or as replacements for those that have been withdrawn from use.

## Materials and Methods

The test compounds tetrabutylammonium tetrafluoroborate [TBA][BF_4_] (99% purity), tetrahexylammonium tetrafluoroborate [THA][BF_4_] (≥97% purity), tetrabutylammonium hexafluorophosphate [TBA][PF_6_] (98% purity) and tetrahexylammonium hexafluorophosphate [THA][PF_6_] (≥97% purity) used in the study were purchased from Sigma–Aldrich Chemical Co (Poznań, PL).

A pot experiment for the determination of potential phytotoxicity of the QASs was carried out in the vegetation hall of the Department of Biochemistry and Ecotoxicology at Jan Długosz University in Częstochowa, PL. The same weights of seeds were sown to plastic pots with a diameter of 90 mm containing 250 g of soil. The soil used in the experiment was light loam with dissolved matter content of approx. 10%, organic carbon content of 0.9% and pH equal to 6.0. Three weeks after emergence, the plants were sprayed with solutions of the examined compounds. Weighed amounts of each QAS were dissolved in 10 mL of a water–methanol mixture to yield 0.5%, 1.0% and 2.0% concentrations. Then, 2 mL of the solution with a given concentration was collected and used for spraying leaves of plants growing in the pots. The controls were also prepared in an analogous manner, and they were sprayed with an aqueous-alcoholic solution, but without QAS addition. Thus, the amounts of QASs administered to the surface were equal to the amounts of active substances contained in commercial herbicides. This allowed to a certain extent for the comparison of phytotoxicity of the studied QASs with substances available in the market.

Throughout the testing period (21 days), constant substrate moisture content at the level required for the plants (70% field water capacity), a constant temperature of 20 ± 2 °C and a light intensity of 160 μmol m^−2^ s^−1^ were maintained in the system of 16 h/day and 8 h/night.

The tests concerning determination of an effect of salts applied to soil on selected weed species were conducted for 28 days under the same conditions as in the case of foliar examinations. The examined compounds were added to the soil (incorporation) and weed seeds of *G. parviflora, C. album* and *R. acetosa*, sown on such prepared ground. The concentrations for each QAS compound were 100, 400 and 700 mg kg^−1^ of soil dry weight. The analytically weighed amounts of QASs were dissolved in 10 mL acetone, and then 50 g of quartz sand was added, rinsing the dish with pure acetone several times. In order to obtain the same QAS concentration from each substrate volume, after the open air evaporation of acetone, the weighed amount of quartz sand was carefully mixed with 200 g of soil. For each concentration, three independent samples were prepared. Identical weights of seeds of the studied plant species were immediately sown into the substrates thus prepared.

Phytotoxicity of the QAS compounds was determined by measuring dry weight content of the weed leaves, shoot length and root length. Shoot and root lengths were measured as described by Wang et al. (2009). Shoot length can be defined as the length from the tip of the longest leaf to the base of culms, and root length can be defined as the length from the tip of the longest root to the root-shoot junction. Inhibition ratio was calculated as (length in control group—length in treatment group) x 100%/length control group. Results were expressed as shoot height and root length inhibition in comparison to control.

Photosynthetic pigments content was determined according to the method reported by Oren et al. (1993). Fresh leaves (0.2 g) homogenized in 20 mL 80% acetone using a mortar and pestle were placed into a centrifuge tube. The extraction was carried out in darkness for 24 h, and the extracts were centrifuged for 10 min. The supernatants were used for determination of the content of chlorophyll *a* and *b*, and carotenoids by measuring absorbance at 470, 647 and 664 nm, respectively. Pigment content was expressed as mg g^−1^ dry weight (DW).

Plant dry weights were measured as described by Kowalska (2004). One gram fresh weight samples of the plants were dried to constant weight at a temperature of 105°C. The dry weight content was expressed as g g^−1^ fresh weight (FW).

The results were analyzed statistically using a statistical software package Statistica v. 12.0 (Statsoft, Inc., Krakow, PL). The data from three measurements (n = 3) were analyzed using one-way ANOVA. Homogeneous groups were calculated using Tukey’s test with *p* < *0.05*. The results were expressed as the mean ± standard deviation.

## Results and Discussion

**Fig. 1:**
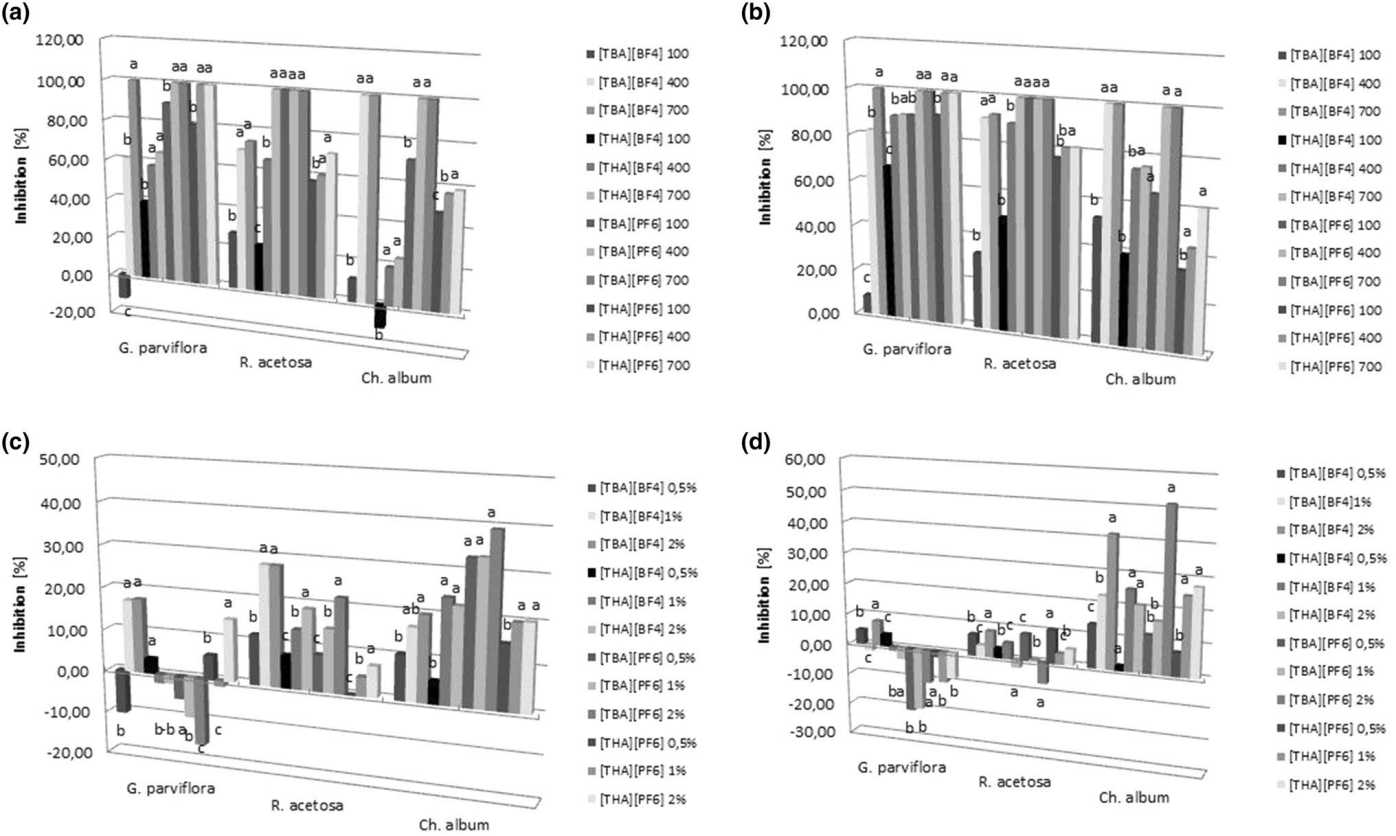
Inhibition of growth plant (**a**) and root (**b**) *G. parviflora, R. acetosa* and *C. album* exposed to [TBA][BF_4_], [THA][BF_4_], [TBA][PF_6_] and [THA][PF_6_] in soil and inhibition of growth plant (**c**) and root (**d**) weeds exposed to QASs applied as foliar spraying (n = *3*). Values denoted by the same letters in the concentrations do not differ statistically at *p* < *0.05*

The results showed that the examined QASs exhibited herbicidal properties. The strongest herbicidal activity was observed for all compounds after the salts were introduced into the soil. The use of [TBA][BF_4_], [THA][BF_4_], [TBA][PF_6_] and [THA][PF_6_] in the form of soil application caused a marked inhibition of the growth of plants and their roots, increasing with increased concentration of QAS application. *C. album* was the most sensitive to QASs among the species tested. None of *C. album* seeds germinated following application of [TBA][BF_4_] and [TBA][PF_6_] (Fig. 1a, b). In turn, foliar spraying caused an inhibition in the growth of aerial parts and roots of *C. album*. Observed changes in the cases of QAS soil applications were positively correlated with applied concentrations of these compounds. Spraying of *R. acetosa* leaves with solutions of [TBA][BF_4_], [THA][BF_4_], [TBA][PF_6_] resulted in an inhibition of aerial parts growth, depending on compound concentration, but no such effect on the length of the roots of this species was noted (less than 10% or no inhibition). The most resistant plant to foliar treatment was *G. parviflora*, for which only slight growth inhibition was observed following spray applications of 1% and 2% solutions of [TBA][BF_4_] and 2% solution of [THA][PF_6_]. In the cases of other concentrations of these QASs and after an application of [THA][BF_4_] and [TBA][PF_6_], no inhibition in aerial parts growth of *G. parviflora* plants and their roots was observed (Fig. 1c, d).

Biczak et al. (2015) reported extreme phytotoxic effects in common radish and spring barley when tetrafluoroborates with the alkilimidazole cation were added to soil. Additionally this impact depended on the length of carbon chain in the substituent. Chiral ionic liquids used by these authors in the form of foliar spraying of *G. parviflora, C. album* and *R. acetosa* plants also demonstrated an inhibitory effect of these salts on the growth and development of the examined weed species. Salts containing PF_6_^−^ anion in their structure are also considered in the literature as compounds exhibiting high toxicity to plants due to the fact that their hydrolysis produces fluoride ions which are toxic and highly undesirable in the environment (Biczak et al. 2014; Cho et al. 2008; Matzke et al. 2007). Telesiński and Śnioszek (2009) found that fluoride effects on plants concern, inter alia, the negative impact of this element on assimilation processes and photosynthesis, which in turn leads to plant growth inhibition. These phenomena result from the destructive effect of fluorine on chloroplasts. The strong effects of the examined QAS compounds when added to soil may be related to the fact that soil is the environment for plant development, from which they collect water and nutrients, as well as toxic chemicals (Chapman et al. 2012). Peric et al. (2014), Biczak (2016) and Pawłowska and Biczak (2016) reported that the presence of harmful substances in the soil can also cause an inhibition in seed germination, and even complete blockage of their germination capacity.

**Fig. 2:**
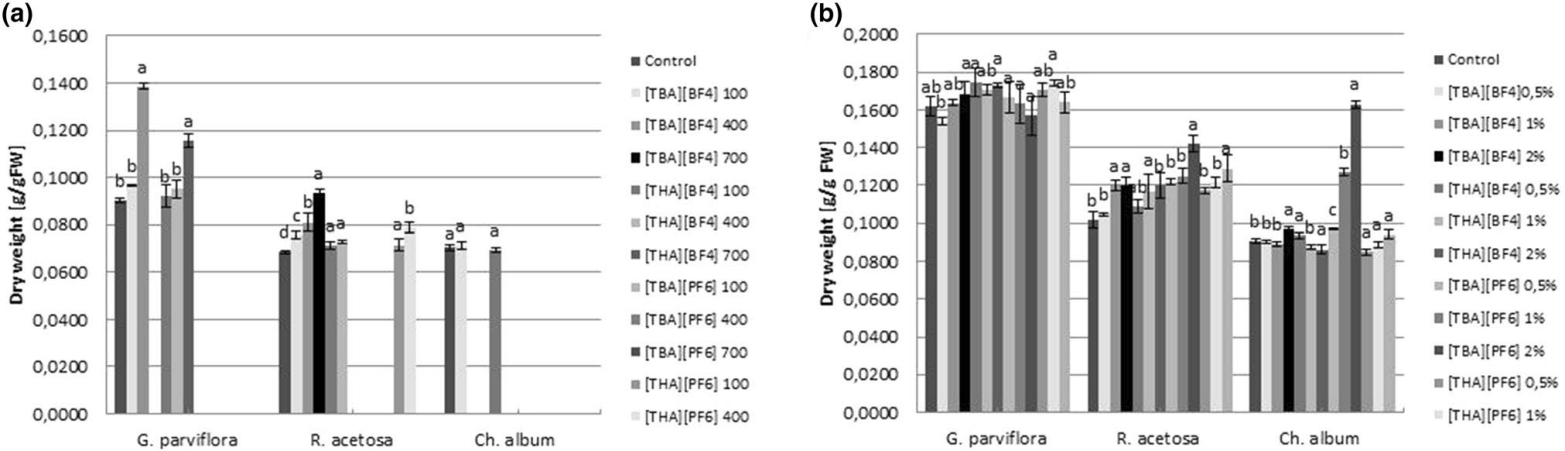
Effect of [TBA][BF_4_], [THA][BF_4_], [TBA][PF_6_] and [THA][PF_6_] in soil (**a**) and spraying (**b**) on dry weight (g g^−1^ FW) weeds (mean ± SD, n = *3*). Values denoted by the same letters in the concentrations do not differ statistically at *p* < *0.05*

Dry weight content of leaves of weeds was also evaluated in the present study. Soil application of QASs led to an increase in dry weight content in the leaves of *G. parviflora* and *R. acetosa* compared to the control. However, foliar spraying of the weeds resulted in an increase in leaf dry weight content only for *R. acetosa*, which increased in a dose dependent fashion. An increase in dry weight level in *C. album* leaves was only found following spraying with 1% and 2% solutions [TBA][PF_6_] (Fig. 2).

An increase in dry weight content in the plants coming into contact with chemicals, including QASs and ILs, was also reported by Biczak et al. (2015) and Matusiak et al. (2013). The results obtained by Liu et al. (2015a), are in contrast to the findings obtained in this study, since the authors observed a decrease in dry weight content in the leaves of broad bean under an influence of 1-butyl-3-methylimidazolium chloride. The discrepancy in the results of the research concerning chemicals effect on dry weight accumulation in plants may prove that the changes in dry weight content are related to, inter alia, species differences of the examined plants.

Changes in photosynthetic pigment content are among the most important biomarkers of oxidative stress in plants. Some authors (Ma et al. 2010; Zhang et al. 2013; Liu et al. 2015b; Herman et al. 1998; Wang et al. 2009) reported almost a linear decline in assimilation pigments with content with increasing levels of QAS or IL compounds in the soil. In the present experiment, the use of all QASs in the form of soil application and foliar spraying caused a reduction in the content of chlorophyll *a*, chlorophyll *b*, total chlorophyll and carotenoids in most of the examined weeds. In turn, soil treatments also resulted in a decline in the ratio of chlorophyll *a* to *b* in *R. acetosa* and *G. apviflora*, as well as a decrease in the ratio of total chlorophyll to carotenoids *G. parviflora* and *C. album*. No such relationships were observed with foliar treatment (Tables 1, 2).

**Table 1:** Effect of QAS compounds in soil on photosynthetic pigments in weed leaves (mean ± SD, *n* = *3*).

Concentration of QASs (mg kg^−1^ soil DW)		Pigments (mg g^−1^ FW)					
		Chl*a*	Chl*b*	Car	Chl*a* + Chl*b*	Chl*a*/Chl*b*	Chl*a* + *b*/car
Gallant soldier (Galinsoga parviflora Cav.)							
	0	1.090 ± 0.035^a^	0.344 ± 0.007^a^	0.265 ± 0.008^a^	1.434 ± 0.038^a^	3.166 ± 0.092^a^	5.419 ± 0.015^a^
[TBA][BF_4_]	100	1.015 ± 0.028^b^	0.322 ± 0.007^b^	0.255 ± 0.007^a^	1.337 ± 0.035^a^	3.147 ± 0.029^a^	5.241 ± 0.040^ab^
	400	0.626 ± 0.004^c^	0.239 ± 0.004^c^	0.175 ± 0.001^b^	0.865 ± 0.007^b^	2.622 ± 0.026^b^	4.925 ± 0.040^b^
	700	–	–	–	–	–	–
[THA][BF_4_]	100	1.070 ± 0.016^a^	0.332 ± 0.006^a^	0.261 ± 0.005^a^	1.402 ± 0.023^a^	3.220 ± 0.016^a^	5.369 ± 0.025^a^
	400	0.895 ± 0.012^b^	0.306 ± 0.001^b^	0.228 ± 0.003^b^	1.201 ± 0.013^b^	2.922 ± 0.029^b^	5.265 ± 0.036^a^
	700	0.760 ± 0.047^c^	0.277 ± 0.017^b^	0.197 ± 0.011^c^	1.037 ± 0.063^c^	2.739 ± 0.023^c^	5.264 ± 0.028^a^
[TBA][PF_6_]	100	–	–	–	–	–	–
	400	–	–	–	–	–	–
	700	–	–	–	–	–	–
[THA][PF_6_]	100	–	–	–	–	–	–
	400	–	–	–	–	–	–
	700	–	–	–	–	–	–
Common sorrel (*Rumex acetosa* L.)							
	0	0.900 ± 0.001^a^	0.270 ± 0.001^a^	0.218^a^	1.170 ± 0.002^a^	3.335 ± 0.016^a^	5.361 ± 0.018^a^
[TBA][BF_4_]	100	0.743 ± 0.002^b^	0.236 ± 0.002^b^	0.181 ± 0.001^b^	0.979 ± 0.004^b^	3.142 ± 0.015^a^	5.409 ± 0.024^a^
	400	0.737 ± 0.005^b^	0.230 ± 0.006^bc^	0.186 ± 0.001^b^	0.967 ± 0.011^b^	3.204 ± 0.062^a^	5.193 ± 0.050^a^
	700	0.587^c^	0.214 ± 0.002^c^	0.169 ± 0.002^c^	0.801 ± 0.001^c^	2.748 ± 0.023^b^	4.742 ± 0.057^b^
[THA][BF_4_]	100	0.774 ± 0.007^b^	0.242 ± 0.003^b^	0.185 ± 0.001^c^	1.016 ± 0.010^b^	3.195 ± 0.016^a^	5.481 ± 0.052^a^
	400	0.754 ± 0.021^b^	0.269 ± 0.009^a^	0.199 ± 0.006^b^	1.023 ± 0.030^b^	2.807 ± 0.030^b^	5.134 ± 0.012^a^
	700	–	–	–	–	–	–
[TBA][PF_6_]	100	–	–	–	–	–	–
	400	–	–	–	–	–	–
	700	–	–	–	–	–	–
[THA][PF_6_]	100	0.854 ± 0.021^a^	0.279 ± 0.007^a^	0.223 ± 0.005^a^	1.133 ± 0.028^a^	3.063 ± 0.021^b^	5.081 ± 0.006^a^
	400	0.726 ± 0.003^b^	0.218 ± 0.006^b^	0.187 ± 0.004^b^	0.944 ± 0.007^b^	3.326 ± 0.092^a^	5.042 ± 0.110^a^
	700	–	–	–	–	–	–
White goosefoot (*Chenopodium album* L.)							
	0	0.742 ± 0.023^a^	0.225 ± 0.007^a^	0.188 ± 0.005^a^	0.967 ± 0.029^a^	3.305 ± 0.027^a^	5.152 ± 0.016^a^
[TBA][BF_4_]	100	0.679 ± 0.018^b^	0.193 ± 0.007^b^	0.172 ± 0.002^b^	0.872 ± 0.024^b^	3.520 ± 0.031^a^	5.072 ± 0.078^a^
	400	–	–	–	–	–	–
	700	–	–	–	–	–	–
[THA][BF_4_]	100	0.701 ± 0.006^a^	0.212 ± 0.006^a^	0.189 ± 0.001^a^	0.913 ± 0.011^a^	3.304 ± 0.064^a^	4.833 ± 0.080^b^
	400	–	–	–	–	–	–
	700	–	–	–	–	–	–
[TBA][PF_6_]	100	–	-	–	–	–	–
	400	–	–	–	–	–	–
	700	–	–	–	–	–	–
[THA][PF_6_]	100	–	–	–	–	–	–
	400	–	–	–	–	–	–
	700	–	–	–	–	–	–

Values denoted by the same letters in the columns do not differ statistically at *p* < *0.05*

*Chla* chlorophyll *a, Chlb* chlorophyll *b, Chla* + *Chlb* chlorophyll *a* + chlorophyll *b, car* carotenoides, *Chla*/*Chlb* chlorphyll *a*/chlorophyll *b, Chla* + *b*/*car* (chlorophyll *a* + chlorophyl *b*)/carotenoides

**Table 2:** Effect of QAS compounds sprayed with solutions on photosynthetic pigments in weed leaves (mean ± SD, n = *3*).

Concentration of QASs (mg kg^−1^ soil DW)		Pigments (mg g^−1^ FW)					
		Chl*a*	Chl*b*	Car	Chl*a* + Chl*b*	Chl*a*/Chl*b*	Chl*a* + *b*/car
Gallant soldier (Galinsoga parviflora Cav.)							
	0	0.694 ± 0.004^b^	0.224 ± 0.003^b^	0.205 ± 0.001^b^	0.918 ± 0.006^b^	3.100 ± 0.024^b^	4.484 ± 0.051^a^
[TBA][BF_4_]	100	0.761 ± 0.016^a^	0.243 ± 0.005^a^	0.219 ± 0.004^a^	1.004 ± 0.021^a^	3.129 ± 0.004^b^	4.952 ± 0.013^a^
	400	0.614 ± 0.019^c^	0.180 ± 0.006^c^	0.178 ± 0.004^c^	0.794 ± 0.014^c^	3.421 ± 0.211^a^	4.460 ± 0.015^a^
	700	0.607 ± 0.009^c^	0.200 ± 0.003^c^	0.187 ± 0.002^c^	0.807 ± 0.012^c^	3.044 ± 0.009^b^	4.304 ± 0.026^a^
[THA][BF_4_]	100	0.688 ± 0.016^a^	0.231 ± 0.008^a^	0.212 ± 0.004^a^	0.919 ± 0.024^a^	2.982 ± 0.039^b^	4.339 ± 0.045^ab^
	400	0.597 ± 0.020^b^	0.182 ± 0.009^b^	0.182 ± 0.006^b^	0.779 ± 0.030^b^	3.277 ± 0.062^a^	4.284 ± 0.032^ab^
	700	0.555 ± 0.013^b^	0.183 ± 0.006^b^	0.179 ± 0.004^b^	0.739 ± 0.019^b^	3.034 ± 0.029^ab^	4.120 ± 0.022^b^
[TBA][PF_6_]	100	0.606 ± 0.002^b^	0.204 ± 0.001^b^	0.184 ± 0.001^b^	0.810 ± 0.003^b^	2.966 ± 0.010^a^	4.411 ± 0.018^a^
	400	0.607 ± 0.007^b^	0.206 ± 0.004^b^	0.183 ± 0.002^b^	0.813 ± 0.011^b^	2.954 ± 0.037^a^	4.435 ± 0.037^a^
	700	0.580 ± 0.008^b^	0.195 ± 0.004^b^	0.179 ± 0.002^b^	0.775 ± 0.012^b^	2.968 ± 0.020^a^	4.339 ± 0.028^a^
[THA][PF_6_]	100	0.580 ± 0.006^b^	0.198 ± 0.001^b^	0.180 ± 0.002^b^	0.778 ± 0.005^b^	2.933 ± 0.036^b^	4.326 ± 0.026^ab^
	400	0.464 ± 0.005^c^	0.157 ± 0.002^c^	0.154 ± 0.001^c^	0.620 ± 0.005^c^	2.957 ± 0.049^b^	4.041 ± 0.030^b^
	700	0.422 ± 0.010^c^	0.124 ± 0.003^d^	0.138 ± 0.004^d^	0.546 ± 0.013^d^	3.406 ± 0.042^a^	3.969 ± 0.028^b^
Common sorrel (*Rumex acetosa* L.)							
	0	0,843 ± 0.007^a^	0.260 ± 0.003^b^	0.207 ± 0.002^b^	1,103 ± 0.005^a^	3.236 ± 0.058^a^	5.319 ± 0.053^a^
[TBA][BF_4_]	100	0.839 ± 0.004^a^	0.277 ± 0.001^a^	0.226 ± 0.001^a^	1.116 ± 0.005^a^	3.034 ± 0.008^ab^	4.926 ± 0.013^ab^
	400	0.769 ± 0.006^b^	0.258 ± 0.005^b^	0.205 ± 0.003^b^	1.027 ± 0.003^b^	2.983 ± 0.078^b^	5.019 ± 0.048^ab^
	700	0.673 ± 0.001^c^	0.231 ± 0.002^c^	0.187 ± 0.001^c^	0.904 ± 0.003^c^	2.914 ± 0.026^b^	4.845 ± 0.035^a^
[THA][BF_4_]	100	0.736 ± 0.002^b^	0.236 ± 0.002^b^	0.202 ± 0.001^a^	0.972 ± 0.002^b^	3.121 ± 0.026^ab^	4.822 ± 0.026^b^
	400	0.699 ± 0.011^b^	0.233 ± 0.008^b^	0.184 ± 0.004^b^	0.931 ± 0.005^b^	3.006 ± 0.146^b^	5.050 ± 0.084^ab^
	700	0.636 ± 0.006^c^	0.194 ± 0.003^c^	0.171^b^	0.830 ± 0.009^c^	3.283 ± 0.030^a^	4.863 ± 0.048^b^
[TBA][PF_6_]	100	0.672 ± 0.054^b^	0.216 ± 0.018^b^	0.178 ± 0.015^b^	0.888 ± 0.072^b^	3.111 ± 0.007^ab^	4.979 ± 0.019^ab^
	400	0.661 ± 0.021^b^	0.221 ± 0.008^b^	0.182 ± 0.006^b^	0.882 ± 0.030^b^	2.992 ± 0.019^b^	4.848 ± 0.042^b^
	700	0.689 ± 0.004^b^	0.213 ± 0.004^b^	0.184 ± 0.006^b^	0.901 ± 0.006^b^	3.242 ± 0.067^a^	4.892 ± 0.129^b^
[THA][PF_6_]	100	0.767 ± 0.002^b^	0.254 ± 0.001^a^	0.200 ± 0.001^a^	1.021 ± 0.002^b^	3.024 ± 0.016^a^	5.098 ± 0.027^a^
	400	0.765 ± 0.005^b^	0.247 ± 0.002^a^	0.199 ± 0.003^a^	1.012 ± 0.004^b^	3.094 ± 0.046^a^	5.076 ± 0.095^a^
	700	0.694 ± 0.017^c^	0.246 ± 0.008^a^	0.186 ± 0.002^b^	0.940 ± 0.025^c^	2.828 ± 0.020^b^	5.053 ± 0.182^a^
White goosefoot (*Chenopodium album* L.)							
	0	1.259 ± 0.012^a^	0.336 ± 0.005^a^	0.303 ± 0.003^a^	1.595 ± 0.018^a^	3.743 ± 0.027^a^	5.268 ± 0.016^a^
[TBA][BF_4_]	100	1.133 ± 0.005^b^	0.306 ± 0.002^b^	0.287 ± 0.001^ab^	1.439 ± 0.007^b^	3.708 ± 0.017^a^	5.011 ± 0.038^a^
	400	1.085 ± 0.019^b^	0.288 ± 0.007^c^	0.274 ± 0.005^b^	1.373 ± 0.026^b^	3.771 ± 0.028^a^	5.015 ± 0.016^a^
	700	0.889 ± 0.005^c^	0.235 ± 0.002^d^	0.228 ± 0.004^c^	1.124 ± 0.006^c^	3.778 ± 0.021^a^	4.926 ± 0.091^a^
[THA][BF_4_]	100	1.093 ± 0.006^b^	0.300 ± 0.001^b^	0.268 ± 0.003^b^	1.393 ± 0.005^b^	3.649 ± 0.026^a^	5.189 ± 0.031^a^
	400	1.021 ± 0.006^bc^	0.276 ± 0.005^bc^	0.254 ± 0.001^b^	1.298 ± 0.011^bc^	3.700 ± 0.044^a^	5.108 ± 0.041^a^
	700	0.967 ± 0.033^c^	0.261 ± 0.012^c^	0.247 ± 0.008^b^	1.228 ± 0.045^c^	3.708 ± 0.048^a^	4.963 ± 0.033^a^
[TBA][PF_6_]	100	1.026 ± 0.025^b^	0.292 ± 0.011^b^	0.242 ± 0.006^b^	1.318 ± 0.034^b^	3.522 ± 0.067^a^	5.454 ± 0.061^a^
	400	0.850 ± 0.006^c^	0.256 ± 0.004^c^	0.209 ± 0.001^c^	1.106 ± 0.009^c^	3.321 ± 0.032^b^	5.291 ± 0.026^a^
	700	0.789 ± 0.005^c^	0.230 ± 0.004^d^	0.196 ± 0.001^c^	1.019 ± 0.009^c^	3.431 ± 0.035^ab^	5.212 ± 0.037^a^
[THA][PF_6_]	100	1.195 ± 0.011^b^	0.308 ± 0.003^b^	0.258 ± 0.003^b^	1.503 ± 0.014^ab^	3.881 ± 0.010^a^	5.826 ± 0.019^a^
	400	1.206 ± 0.043^b^	0.332 ± 0.012^a^	0.268 ± 0.009^b^	1.539 ± 0.055^a^	3.631 ± 0.002^a^	5.736 ± 0.028^a^
	700	1.109 ± 0.013^c^	0.308 ± 0.004^b^	0.255 ± 0.003^b^	1.416 ± 0.017^b^	3.604 ± 0.013^a^	5.555 ± 0.021^ab^

Values denoted by the same letters in the columns do not differ statistically at *p* < *0.05*

*Chla* chlorophyll *a, Chlb* chlorophyll *b, Chla* + *Chlb* chlorophyll *a* + chlorophyll *b, car* carotenoides, *Chla*/*Chlb* chlorphyll *a*/chlorophyll *b, Chla* + *b*/*car* (chlorophyll *a* + chlorophyl *b*)/carotenoides

It has been reported that chemicals in the soil have caused oxidative stress in plants, and that overproduction of reactive oxygen species (ROS) can damage, inter alia, membranes of chloroplasts (Sun et al. 2007; Gengmao et al. 2015; Arias-Baldrich et al. 2015). This causes disorders in chlorophyll synthesis (mainly chlorophyll *a*) and premature plant aging. A decrease in the content of carotenoids found in this study, which are the primary line of defence of photosystems PSI and PSII against ROS, can also result in harmful effects of the examined QASs on the weeds (Biczak 2016; Biczak et al. 2016; Pawłowska and Biczak 2016).

In summary, [TBA][BF_4_], [THA][BF_4_], [TBA][PF_6_] and [THA][PF_6_] showed selective herbicidal properties for *G. parviflora, C. album* and *R. acetosa*. Phytotoxicity of QASs to the highest degree depended on the form of the applied treatment. The strongest herbicidal activity was found for the compounds applied to the soil in which the weed seeds were sown. However, no major changes in growth and development of the weeds were observed with spraying applications. With both forms of treatment, QAS effects were positively correlated with the concentration of the compound, i.e., higher concentrations resulted in greater effects. An important factor affecting the phytotoxicity of examined salts was also the type of anion and the length of alkyl substituent. Compounds showing higher toxic activity for weeds were QAS compounds with the PF_6_^−^ anion. Also, higher phytotoxicity was observed for QAS compounds with substituents containing four carbon atoms. Moreover, an effect of the examined compounds depended on plant species. *C. album* was the most sensitive species, as evidenced by complete inhibition of shoot and root growth. The weeds in which changes in external appearance and an inhibition of growth and roots length were observed also showed changes in dry weight content. A decrease in levels of assimilation pigments was positively correlated with an increase in QAS concentration in the soil or in the spray solution.

## References

[CR1] Arias-Baldrich C, Bosch N, Begines D, Feria AB, Monreal JA, García-Mauriño S (2015). Proline synthesis in barley under iron deficiency and salinity. J Plant Physiol.

[CR2] Biczak R (2016). Quaternary ammonium salts with tetrafluoroborate anion: phytotoxicity and oxidative stress in terrestrial plants. J Hazard Mater.

[CR3] Biczak R, Pawłowska B, Bałczewski P, Rychter P (2014). The role of the anion in the toxicity of imidazolium ionic liquids. J Hazard Mater.

[CR4] Biczak R, Pawłowska B, Feder-Kubis J (2015). The phytotoxicity of ionic liquids from natural pool of (–)-menthol with tetrafluoroborate anion. Environ Sci Pollut Res.

[CR5] Biczak R, Telesiński A, Pawłowska B (2016). Oxidative stress in spring barley and common radish exposed to quaternary ammonium salts with hexafluorophosphate anion. Plant Physiol Biochem.

[CR6] Chapman N, Miller AJ, Lindsey K, Whalley WR (2012). Roots, water, and nutrient acquisition: let’s get physical. Trends Plant Sci.

[CR7] Cho Ch-W, Pham TPT, Jeon Y-Ch, Yun Y-S (2008). Influence of anions on the ionic liquids to a phytoplankton *Selenastrum capricornutum*. Green Chem.

[CR8] Gengmao Z, Yu H, Xing S, Shihui L, Quanmei S, Changhai W (2015). Salinity stress increases secondary metabolites and enzyme activity in safflower. Ind Crops Prod.

[CR9] Grabińska-Sota E (2004). Assessment of the impact of quaternary ammonium salts on the aquatic environment.

[CR10] Herman B, Biczak R, Gurgul E (1998). Effect of 1,10-phenanthroline on peroxidase and catalase activity and chlorophyll, sugar, and ascorbic acid contents. Biol Plant.

[CR11] Kowalska I (2004). The content of selected components of spinach (*Spinacia oleracea* L.) grown at varying levels of calcium.

[CR12] Liu H, Zhang S, Zhang X, Chen C (2015). Growth inhibition and effect on photosystem by three imidazolium ionic liquids in rice seedlings. J Hazard Mater.

[CR13] Liu T, Zhu L, Wang J, Wang J, Xie H (2015). The genotoxic and cytotoxic effects of 1-butyl-3-methylimidazolium chloride in soil on *Vicia faba* seedlings. J Hazard Mater.

[CR14] Ma J-M, Cai L-L, Zhang B-J, Hu L-W, Li X-Y, Wang J-J (2010). Acute toxicity and effects of 1-alkyl-3-methylimidazolium bromide ionic liquids on green algae. Ecotoxicol Environ Saf.

[CR15] Matusiak A, Lewkowski J, Rychter P, Biczak R (2013). Phytotoxicity of new furan-derived aminophosphonic acid, N-aryl furaldimines and 5-nitrofuraldimine. J Agric Food Chem.

[CR16] Matzke M, Stolte S, Thiele K, Juffernholz T, Arning J, Ranke J, Welz-Biermann U, Jastorff B (2007). The influence of anion species on the toxicity of 1-alkyl-3-methylimidazolium ionic liquids observed in an (eco)toxicological test battery. Green Chem.

[CR17] Niemczak M, Giszter R, Czerniak K, Marcinkowska K, Walkiewicz F (2015). Bis(ammonium) ionic liquids with herbicidal anions. RSC Adv.

[CR18] Oren R, Werk KS, Buchmann N, Zimmermann R (1993). Chlorophyll-nutrient relationships identify nutritionally caused decline in *Picea abies* stands. Can J For Res.

[CR19] Pawłowska B, Biczak R (2016). Evaluation of the effect of tetraethylammonium bromide and chloride on the growth and development of terrestrial plants. Chemosphere.

[CR20] Peric B, Sierra J, Martí E, Cruañas R, Garau MA (2014). A comparative study of the terrestrial ecotoxicity of selected protic and aprotic ionic liquids. Chemosphere.

[CR21] Pernak J, Niemczak M, Materna K, Żelechowski K, Marcinkowska K, Praczyk T (2016). Synthesis, properties and evaluation of biological activity of herbicidal ionic liquids with 4-(4-chloro-2-methylphenoxy)butanoate anion. RSC Adv.

[CR22] Praczyk T, Skrzypczak G (2004). Herbicides.

[CR23] Sun B, Jing Y, Chen K, Song L, Chen F, Zhang L (2007). Protective effect of nitric oxide on iron deficiency-induced oxidative stress in maize (*Zea mays*). J Plant Physiol.

[CR24] Telesiński A, Śnioszek M (2009). Bioindicators of environmental pollution with fluorine. Bromat Chem Toksykol.

[CR25] Travlos IS, Lysandrou M, Apostolidis V (2014). Efficacy of the herbicide GF-2581 (penoxsulam + florasulam) against broadleaf weeds in olives. Plant Soil Environ.

[CR26] Wang L-S, Wang L, Wang L, Wang G, Li Z-H, Wang J-J (2009). Effect of 1-butyl-3-methylimidazolium tetrafluoroborate on the wheat (*Triticum aestivum* L.) seedlings. Environ Toxicol.

[CR27] Zhang B, Li X, Chen D, Wang J (2013). Effects of 1-octyl-3-methylimidazolium bromide on the antioxidant system of *Lemna minor*. Protoplasma.

